# Distribution of hydrogen sulfide (H_2_S)-producing enzymes and the roles of the H_2_S donor sodium hydrosulfide in diabetic nephropathy

**DOI:** 10.1007/s10157-012-0670-y

**Published:** 2012-08-08

**Authors:** Junichiro Yamamoto, Waichi Sato, Tomoki Kosugi, Tokunori Yamamoto, Toshihide Kimura, Shigeki Taniguchi, Hiroshi Kojima, Shoichi Maruyama, Enyu Imai, Seiichi Matsuo, Yukio Yuzawa, Ichiro Niki

**Affiliations:** 1 Division of Nephrology, Nagoya University, 65 Tsurumai, Showa-ku, Nagoya, Aichi 466-8550 Japan; 2 Division of Urology, Nagoya University, 65 Tsurumai, Showa-ku, Nagoya, Aichi 466-8550 Japan; 3 Department of Pharmacology, Oita University Faculty of Medicine, 1-1 Idaigaoka, Hasama, Yufu, Oita 879-5593 Japan; 4 Division of Nephrology, Internal Medicine, Fujita Health University School of Medicine, 1-98 Kutsukake, Toyoake, Aichi 470-1192 Japan

**Keywords:** Hydrogen sulfide (H_2_S), Cystathionine β-synthase (CBS), Cystathionine γ-lyase (CSE), Diabetic nephropathy

## Abstract

**Background:**

Hydrogen sulfide (H_2_S) has recently been found to play beneficial roles in ameliorating several diseases, including hypertension, atherosclerosis and cardiac/renal ischemia–reperfusion injuries. Cystathionine β-synthase (CBS) and cystathionine γ-lyase (CSE), the main enzymes in the transsulfuration pathway, catalyze H_2_S production in mammalian tissues. However, the distributions and precise roles of these enzymes in the kidney have not yet been identified.

**Methods:**

The present study examined the localization of both enzymes in the normal kidney and the effect of the H_2_S donor sodium hydrosulfide (NaHS) in the renal peritubular capillary (PTC) under conditions of diabetic nephropathy, using pancreatic β-cell-specific calmodulin-overexpressing transgenic mice as a model of diabetes.

**Results:**

In the normal kidney, we detected expression of both CBS and CSE in the brush border and cytoplasm of the proximal tubules, but not in the glomeruli, distal tubules and vascular endothelial cells of renal PTCs. Administration of NaHS increased PTC diameter and blood flow. We further evaluated whether biosynthesis of H_2_S was altered in a spontaneous diabetic model that developed renal lesions similar to human diabetic nephropathy. CSE expression was markedly reduced under diabetic conditions, whereas CBS expression was unaffected. Progressive diabetic nephropathy showed vasoconstriction and a loss of blood flow in PTCs that was ameliorated by NaHS treatment.

**Conclusion:**

These findings suggest that CSE expression in the proximal tubules may also regulate tubulointerstitial microcirculation via H_2_S production. H_2_S may represent a target of treatment to prevent progression of ischemic injury in diabetic nephropathy.

## Introduction

Hydrogen sulfide (H_2_S) has traditionally been considered a toxic gas with the smell of rotten eggs, but is now also known as a third gasotransmitter, along with nitric oxide (NO) and carbon monoxide (CO) [1, 2]. The physiological and pathological functions of H_2_S include neurotransmission [3], vascular relaxation [3, 4], insulin secretion [5], cell proliferation and apoptosis [6].

The key enzymatic production of H_2_S from l-cysteine in mammalian tissues is catalyzed by cystathionine γ-lyase (CSE) and cystathionine β-synthase (CBS) [1, 3]. CSE is distributed in smooth muscle cells, liver and pancreas [5, 7, 8], whereas CBS is found in the brain, liver, kidney and pancreas [5, 8]. However, the roles of these two enzymes in the kidney remain unclear.

We previously reported that hyperglycemia reduces the level of endothelial NO synthase (eNOS) expression in the diabetic kidney, thereby reducing NO production and subsequently inducing endothelial dysfunction [9]. We postulated that hyperglycemia would also decrease CSE expression in the kidney, which may cause renal microcirculation injury and renal ischemia. To investigate the roles of CSE and CBS in the kidney, the present study examined the localization of both enzymes in the normal kidney and the effect of the H_2_S donor sodium hydrosulfide (NaHS) in the renal peritubular capillary (PTC) under conditions of diabetic nephropathy, using pancreatic β-cell-specific calmodulin-overexpressing transgenic (CaMTg, also called as OVE26) mice as a model of diabetes [9].

## Materials and methods

### Animals

CaMTg mice were kindly provided by Prof. A. R. Means (Duke University, Durham, NC, USA). Male transgenic mice were bred with female ICR strain mice (Japan SLC, Hamamatsu, Japan). The methods for the production and the phenotypes of CaMTg mice have been described previously [9–11]. Briefly, they show spontaneous hyperglycemia at 4 weeks old, resulting in advanced lesions at 3 months old. Non-transgenic (nTg) littermates were used as normal controls. Blood glucose levels were measured monthly, using a glucometer. These mice were killed at 3 months old. Blood urea nitrogen, serum creatinine and albuminuria were measured as described previously [9]. This study was approved by the Committees on Animal Experiments of Oita University and Nagoya University.

### Renal histology

Kidneys were fixed in 10 % buffered formalin, embedded in paraffin and cut into 4-μm sections. Consecutive sections were stained with a rabbit anti-CSE antibody, a rabbit anti-CBS antibody, a mouse monoclonal anti-angiotensin-converting enzyme (ACE) (CD143) antibody (Chemicon International, Temecula, CA, USA) or a polyclonal sheep anti-human Tamm Horsefall glycoprotein (THP) antibody (Serotec, Oxford, UK). CSE and CBS antibodies were raised in rabbits by injecting the respective peptides (C-L-D-R-A-L-K-A-A-H–P for CSE; C-L-L-A-P–V-A-A-G-G-A for CBS) as previously described [12]. Sections for absorption tests were stained with anti-CSE antibody previously incubated with CSE antigen or with anti-CBS antibody incubated with CBS antigen for 3 h, respectively. Immunoreactive cells were visualized using 3,3′-diaminobenzidine as a substrate. Quantification of immunohistochemical staining was performed in cortical fields, using the MetaMorph 6.3 image analysis computer program (Universal Imaging Co., West Chester, PA, USA).

### Measurements of PTC blood flow velocity, diameter and blood flow

Mice were anesthetized with intraperitoneal pentobarbital sodium at 50 mg/kg body weight (BW). Under anesthesia, the left kidney was exposed via a flank incision. PTC images were obtained using an intravital video CCD camera. The experimental system consisted of a specially-ordered pencil-lens probe videomicroscope with a CCD camera (Nihon Kohden, Tokyo, Japan), a light source (LA-60Me; Hayashi, Tokyo), a monitor (PVM-146 J; Sony, Tokyo) and a videocassette recorder (WV-ST-1; Sony). The probe (diameter, 1 mm) was brought close to the surface of the left kidney for visualization of the PTCs. For the measurement of PTC diameter, images of the vascular segment were rotated to place the segment perpendicular to the scanning line, as described previously [13]. These results were determined by averaging at least five measurements per position during the plateau of the response within 5 min and measuring five positions per mouse (*n* = 5). Blood flow was then derived by multiplying the resulting “blood flow velocity” and “diameter”. Video recording was done by some of the authors and the measurements were separately performed by another author who did not know which study group was examined in a blinded manner.

### In vivo effects of NaHS and saline on the PTC

Male nTg and CaMTg mice at 3 months old were used (*n* = 5 per group). NaHS solution at 56 mg/kg body weight (Sigma-Aldrich, St. Louis, MO, USA) was injected into each animal via a tail vein, and two parameters, PTC blood flow velocity and PTC diameter, were measured within 5 min after treatment, as described above. The dosage used was determined in a previous in vivo experiment [14].

To examine the effects of volume substitution in our first study, we examined values for three parameters with saline administration (the same volume as NaHS treatment) in nTg mice as a second experiment.

### Western blot analysis

Mouse kidney tissues were snap-frozen in liquid nitrogen for protein isolation. Western blot analysis was performed as described previously [15]. The membranes were subsequently incubated overnight at 4 °C with a rabbit anti-CSE antibody, a rabbit anti-CBS antibody or a mouse monoclonal anti-β-actin antibody (Sigma Aldrich). Proteins were visualized with an enhanced chemiluminescence detection system (GE Healthcare UK, Amersham, UK). The intensity of each band was determined using the National Institutes of Health (NIH) Image program.

### Statistical analysis

All values are expressed as the mean ± standard deviation (SD). Statistical analysis was performed using unpaired, two-tailed *t* tests. Values of *P* < 0.05 were taken as indicating a significant difference.

## Results

### CSE and CBS expressions in the nTg kidney

**Fig. 1:**
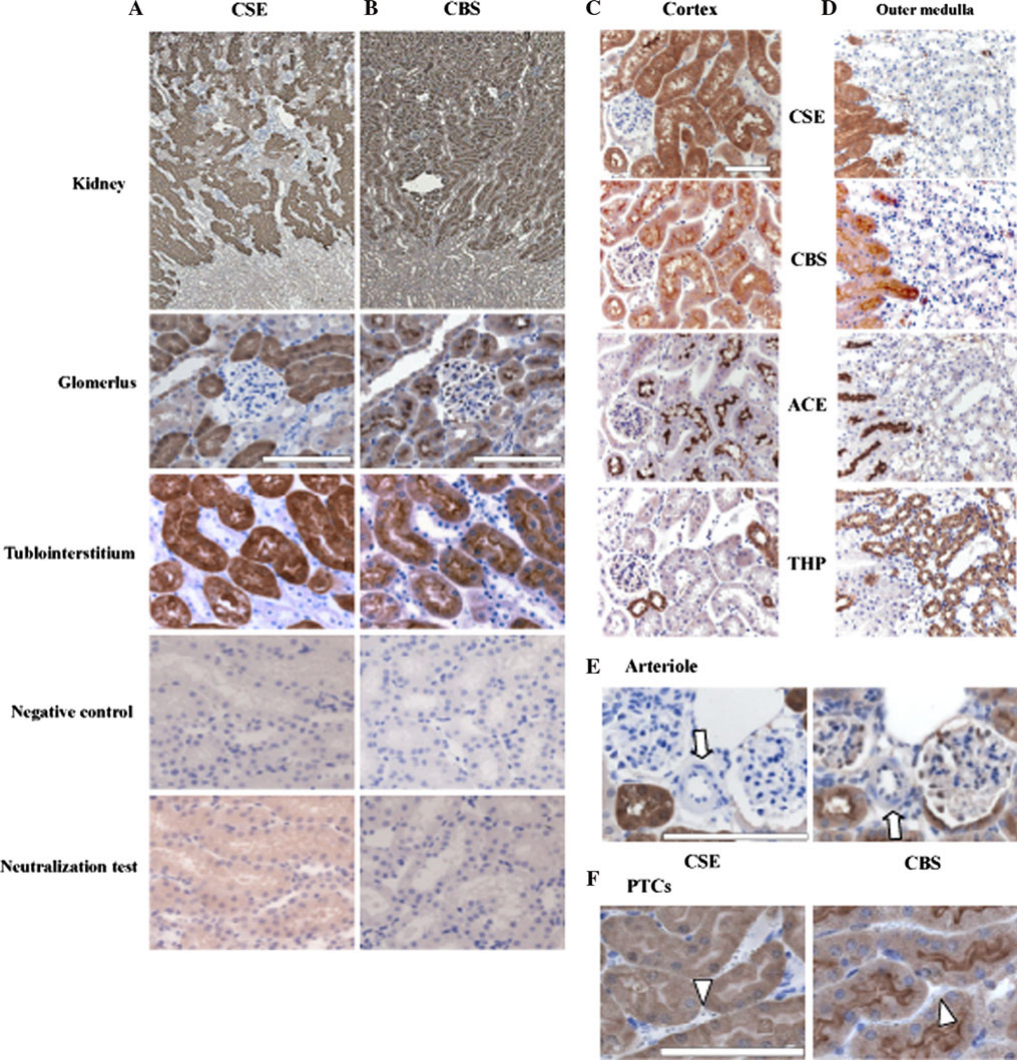
CSE and CBS distributions in the nTg kidney. Immunohistochemistry for CSE (**a**) and CBS (**b**) in the glomeruli and tubulointerstitium of 3-month-old nTg kidney. In the cortex (**c**) and medulla (**d**), immunostainings for CSE, CBS, ACE and THP are shown on consecutive sections. Both CSE and CBS were detected only in the proximal tubules but not in the glomeruli and endothelium. Endothelial cells in arteriole (**e**; *arrow*) and PTCs (**f**; *arrowhead*) did not express CSE and CBS. *Black triangle* proximal tubule, *white triangle* distal tubule. *Bar* 100 μm

CSE and CBS were detected in the cytoplasm and brush border of the proximal tubules, respectively, but expression was detected in neither glomeruli nor distal tubules (Fig. 1a, b). The absorption test did not show any expression (Fig. 1a, b). CSE distribution coincided with the cytoplasm of tubules expressing ACE, a marker of the proximal tubules, but no overlap was seen with the tubules expressing THP, a marker of the ascending limb of Henle’s loop (Fig. 1c, d). Similarly, CBS distribution also coincided with the brush border of the tubules expressing ACE (Fig. 1c, d). Endothelial cells were not found in either renal arterioles or PTCs with CSE or CBS expressions (Fig. 1e, f).

### In vivo effects of NaHS in the normal kidney

**Fig. 2:**
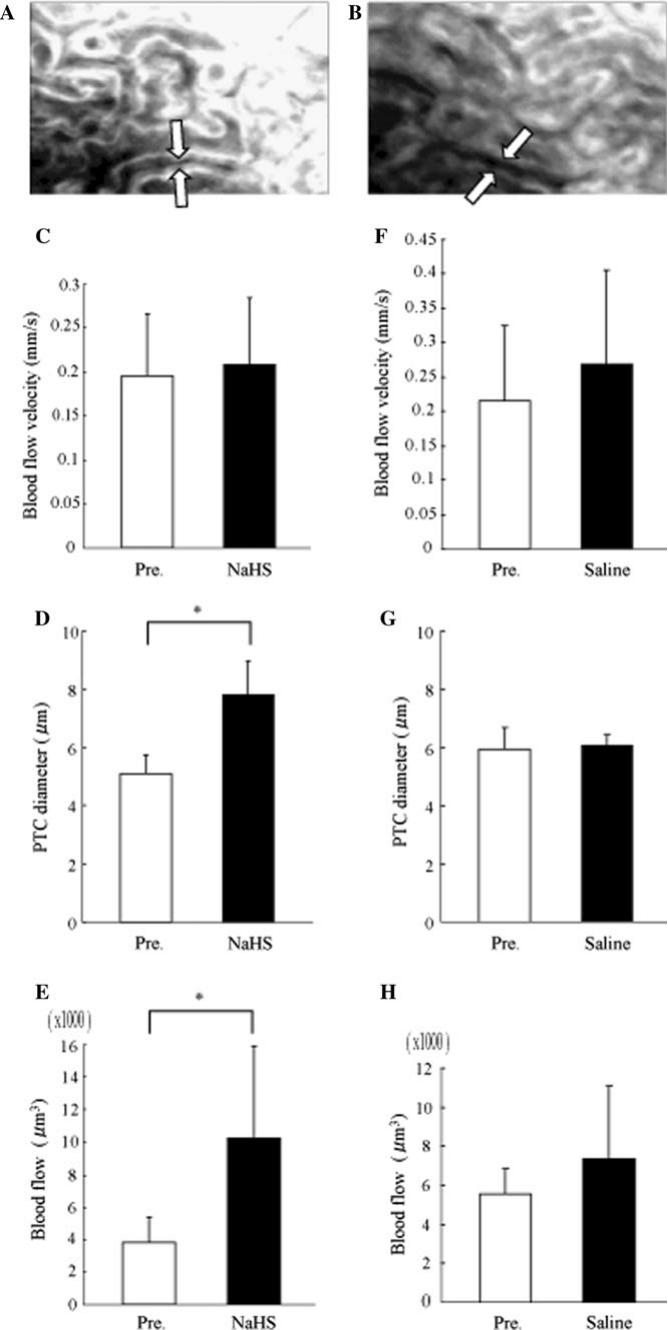
In-vivo effects of NaHS and saline loading in the nTg kidney. PTC images by an intravital video CCD camera at pre- (**a**) and post- (**b**) injections of 56 mg/kg body weight NaHS. The post-injection image was taken 1 min after the NaHS injection. *White arrows* show PTC. While PTC blood flow velocity (**c**) was not affected by the NaHS administration, PTC diameter (**d**) and blood flow (**e**) were increased. On the other hand, isovolume saline loading did not alter PTC blood flow velocity (**f**), its diameter (**g**) and blood flow (**h**). Data are shown as the mean (*columns*) and SD (*bars*). *P < 0.01, *n* = 5

PTC images were taken by an intravital video CCD camera before (Fig. 2a) and after (Fig. 2b) NaHS injection. Although PTC blood flow velocity was unaffected by NaHS administration (Fig. 2c), diameter and blood flow were increased with this treatment (Fig. 2d, e). Systolic blood pressures did not alter following NaHS injection (data not shown). We investigated the effects of the volume substitution in the second experiment. Three parameters were not significantly affected by saline loading (Fig. 2f–h), suggesting that NaHS dilated PTC diameter and increased blood flow.

### CSE and CBS expressions in normal and diabetic kidneys

**Fig. 3:**
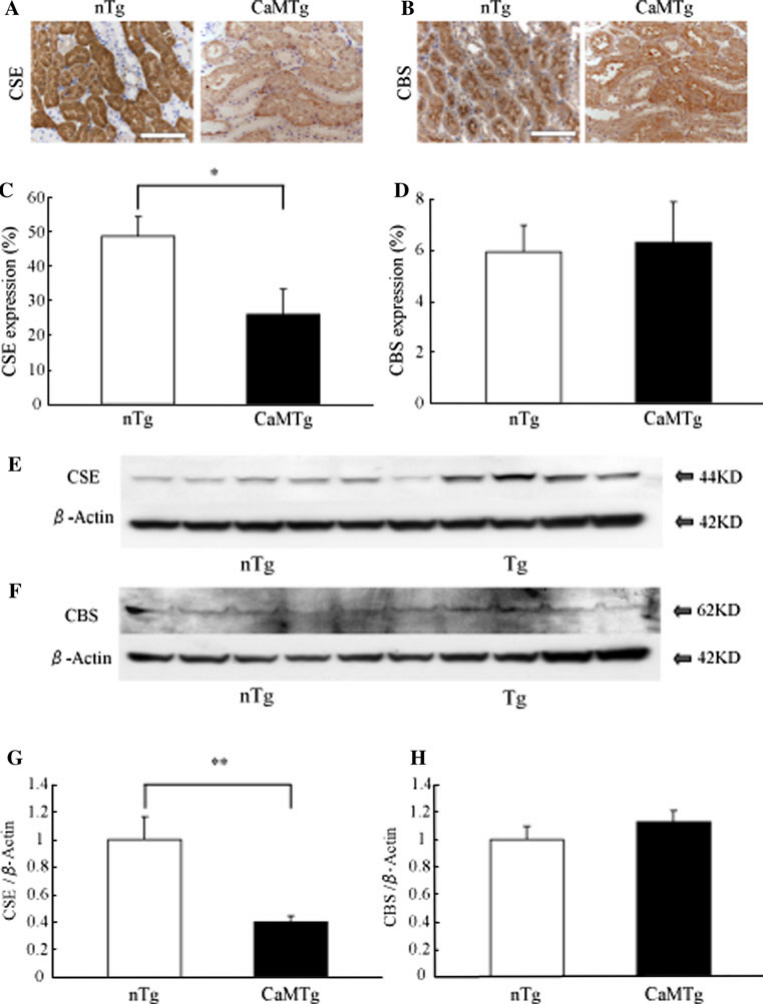
CSE and CBS in the normal and diabetic kidneys. Immnohistochemistry for CSE (**a**) and CBS (**b**) in the nTg and CaMTg tubulointerstitium. Quantitative analysis of immunohistochemistry for CSE (**c**) and CBS (**d**). Western blotting for CSE (**e**) and CBS (**f**). Intensities of CSE (**g**) and CBS (**h**) bands were standardized against those of β-actin. CSE expression was markedly reduced in diabetic mice, whereas CBS expression was not affected. Data are shown as the mean and SD. *P < 0.01, **P < 0.05, *n* = 5. *Bar* 100 μm

CaMTg mice showed higher blood glucose levels at 3 months old (nTg mice, 157 ± 41 mg/dl; CaMTg mice, 657 ± 95 mg/dl) (Table 1). Consistent with the results of the previous report [9], blood urea nitrogen and albuminuria of diabetic CaMTg mice were significantly higher than those of nTg mice. CSE expression was reduced in proximal tubules of CaMTg mice compared to those of the nTg kidney (Fig. 3a, c). Similarly, Western blotting revealed CSE expression was markedly reduced in the diabetic kidney (Fig. 3e, g). In contrast, CBS expression was unaffected in the proximal tubules of diabetic kidneys (Fig. 3b, d, f, h).

**Table 1:** Biochemical parameters

	Non-transgenic mice	CaM-transgenic mice
Body weight (g)	40.4 ± 3.7	36.7 ± 4.8
Blood sugar (mg/dl)	157 ± 41	657 ± 95*
BUN (mg/dl)	22.7 ± 3.0	37.2 ± 4.1*
s-Cre (mg/dl)	0.09 ± 0.04	0.13 ± 0.05
Albuminuria (μg/day)	31 ± 4.0	117 ± 13*

*BUN* blood urea nitrogen, *s*-*Cre* serum creatinine

* *P* < 0.01, *n* = 5

### Impaired renal microcirculation in the tubulointerstitium of the diabetic kidney

**Fig. 4:**
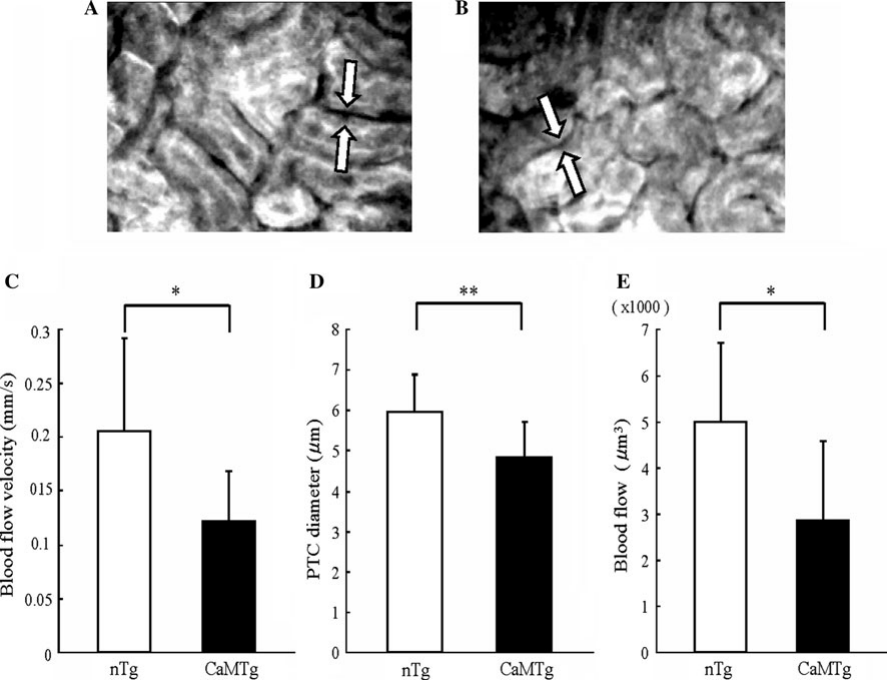
PTC blood flow velocity, diameter and blood flow volume in the nTg and CaMTg kidneys. PTC images by an intravital video CCD camera in nTg (**a**) and CaMTg (**b**) mice without treatment. PTC blood flow velocity (**c**), diameter (**d**) and blood flow (**e**) were all decreased in the CaMTg kidney (*n* = 5). Data are shown as the mean and SD. *P < 0.01, **P < 0.05

PTC images by CCD camera are shown for nTg (Fig. 4a) and CaMTg kidneys (Fig. 4b). PTC blood flow velocity, diameter and blood flow were decreased in the diabetic kidney (Fig. 4c–e). The hematocrit of diabetic CaMTg mice tended to be lower than that of nTg mice (CaMTg vs. nTg = 52.6 ± 2.6 vs. 56.8 ± 4.0 %; *P* = 0.055), suggesting that the diabetic mice in this study were not under greater volume depletion caused by diuresis. In addition, anesthesia also unaffected the hematocrit values in both non-diabetic and diabetic mice (data not shown).

### In vivo effects of NaHS in the CaMTg kidney

**Fig. 5:**
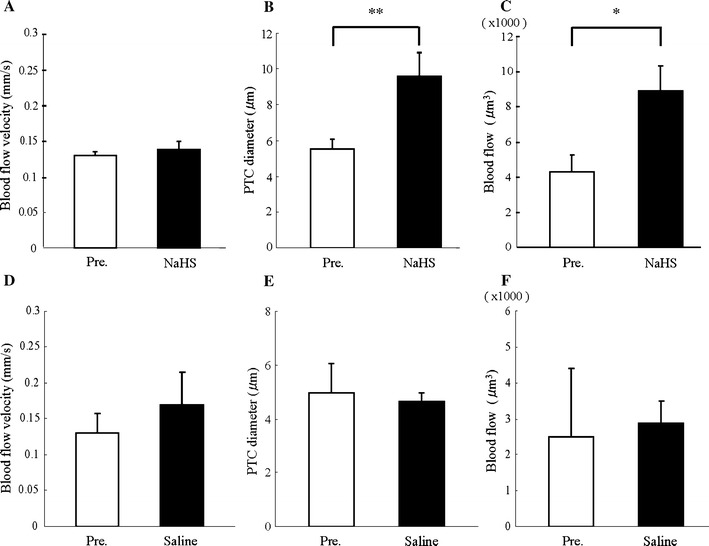
In-vivo effects of NaHS and saline loading in the CaMTg kidney. PTC blood flow velocity (**a**), diameter (**b**) and blood flow (**c**) were measured at pre- and post-injection of NaHS to the CaMTg mice. PTC diameter and blood flow, but not PTC blood flow velocity, were increased by the NaHS treatment. These parameters (**d**–**f**) were not affected by isovolume saline in CaMTg mice. Data are shown as the mean and SD. *P < 0.01, **P < 0.05, *n* = 4

NaHS administration to diabetic mice significantly increased the PTC diameter and blood flow, but not PTC blood flow velocity (Fig. 5a–c). These data are consistent with the pattern of nTg mice treated using NaHS. Similar to nTg mice, systolic and diastolic blood pressures of CaMTg mice also remained unchanged by NaHS injection (data not shown). Treatment of CaMTg mice with isovolume saline did not affect these parameters (Fig. 5d–f).

## Discussion

We detected CSE and CBS in the proximal tubules, but not in the glomeruli or distal tubules, and NaHS administration was found to increase PTC blood flow and PTC diameter using a reliable CCD system. Importantly, CSE expression was markedly decreased in the diabetic kidney with advanced lesions, whereas CBS expression was unaffected. Progressive diabetic nephropathy caused vasoconstriction and a loss of blood flow, which was ameliorated by NaHS treatment. These findings suggest that CSE might regulate PTC microcirculation in the tubulointerstitium and play a key role in the development of advanced diabetic nephropathy.

H_2_S protects several tissues from various types of cell damage through anti-atherosclerotic effects and preservation of mitochondrial function [16, 17]. However, the precise roles of H_2_S in the kidney remain unknown. CSE expression is also reported to be predominant in the vascular smooth muscle cells (VSMCs) [18], although earlier studies did not detect CSE expression in endothelial cells [4, 19]. In our study, however, CSE expression was not found on VSMCs in PTCs and renal arterioles. In the renal system, PTCs are composed only of endothelial cells and play important roles in reabsorption and secretion between blood and the inner lumen of the nephron through active transport, secondary active transport or transcytosis. Instead of PTCs, tubular epithelial cells may express CSE protein alongside PTCs in the kidney.

Previous studies have reported that H_2_S could induce vasorelaxation by directly opening K_ATP_ channels in VSMCs [4, 20]. Unfortunately, we cannot directly verify in vivo the effect of H_2_S through its measurement and treatment. We therefore demonstrated that administration of the H_2_S donor NaHS increased blood flow by PTC dilation. As VSMCs are not present in PTCs, H_2_S may act on the PTC endothelial cells. In addition, H_2_S may be generated in the proximal tubules, and H_2_S thus produced may reach PTC endothelial cells via membrane permeation. Given our results and the findings from several reports that endogenous H_2_S has protective effects on renal ischemia/reperfusion injury [21], H_2_S produced by CSE and/or CBS in the tubular epithelial cells might have beneficial effects on the tubulointerstitium through anti-apoptotic effects and the regulation of hemodynamics. Anti-apoptotic effects of H_2_S have also been reported in other types of cells [12, 22].

Next, we evaluated whether biosynthesis of H_2_S was altered in spontaneously diabetic CaMTg mice, which exhibit hallmarks of human diabetic nephropathy such as hyalinosis of the afferent and efferent arteries with neovascularization. We have previously reported that hyperglycemia reduces eNOS expression, thereby reducing the level of NO production and subsequently inducing endothelial cell proliferation injury [9]. Upregulation of VEGF and downregulation of NO (‘uncoupling’ of the VEGF–NO axis) result in the progression of extra vessels and of extravasation from immature vessels, leading to the development of diabetic nephropathy. Other investigators have demonstrated that both CSE and CBS activities in the pancreas and liver, as well as plasma H_2_S and l-cysteine levels, are increased in streptozotocin-treated diabetic rats [10, 23]. Intriguingly, CSE expression, like eNOS expression, in the proximal tubules was reduced in that diabetic model. H_2_S also promotes angiogenesis through VEGF signaling pathways such as the PI3 K-Akt pathway [24]. As the biological features of H_2_S resemble those of NO, modulation of H_2_S production might be involved in diabetic tubulointerstitial ischemia. High glucose further induces the CSE expression in the β cells in pancreas, in contrast to the renal proximal tubules [10]. Interestingly, l-cysteine or NaHS suppressed apoptosis in pancreatic islet under diabetic status. Indeed, pretreatment with l-cysteine improved the secretory responsiveness following stimulation with glucose. These suggest that H_2_S may protect β cells from glucotoxicity, eventually leading to the promotion of insulin secretion [5].

We emphasize that the diabetic model used in this study showed decreased PTC blood flow velocity and blood flow, in spite of PTC neovascularization. Endothelial dysfunction attributable to eNOS reduction and insulin deficiency might induce decreased PTC blood flow, resulting in tubulointerstitial ischemia and injury [25, 26]. Eventually, this state would create a vicious cycle such as activation of the renin–angiotensin system. We further demonstrated that NaHS administration increased blood flow by PTC dilation. Indeed, CSE reduction results in decreased H2S formation [18]. These findings suggest that CSE in the proximal tubules may regulate the interstitial microcirculation via H_2_S production. As the sensitivity of PTCs to NaHS was maintained in this diabetic model, H_2_S may offer a useful target for the treatment of diabetic nephropathy.
